# Circadian clocks and adaptive immune function: from mechanisms to therapeutic applications

**DOI:** 10.3389/fimmu.2025.1697854

**Published:** 2025-12-03

**Authors:** Aleksandra Szredzka, Anna Chwastowicz, Joanna Pergoł, Paweł Matryba

**Affiliations:** 1 Department of Immunology, Medical University of Warsaw, Warsaw, Poland; 2 Department of Pediatrics and Nephrology, Medical University of Warsaw, Warsaw, Poland; 3 Doctoral School, Medical University of Warsaw, Warsaw, Poland

**Keywords:** adaptive immunity, circadian rhythm, T cell, B cell, chronotherapy

## Abstract

The circadian clock exerts profound regulatory control over adaptive immunity, particularly affecting T and B lymphocyte development, trafficking, activation, and effector function. These immune cells possess autonomous molecular clocks governed by core genes such as *Bmal1*, *Clock*, *Per1/2*, and *Cry1/2*, which influence transcriptional programs related to receptor expression, cytokine signaling, and metabolism. Circadian regulation shapes the daily oscillation of lymphocyte recirculation through lymphoid organs, modulates antigen responsiveness, and fine-tunes subset differentiation-most notably skewing CD4^+^ T cell fate toward Th1, Th2, Th17, or Treg lineages depending on time-of-day-linked signaling cascades such as mTOR/Akt and RORγt/Nfil3 pathways. Similarly, rhythmic glucocorticoid and catecholamine signaling synchronize peripheral lymphocyte clocks with systemic cues, integrating hormonal and environmental information. Clinically, circadian disruption - whether through genetic mutations, shift work, or chronic stress - has been linked to aberrant lymphocyte function, increased autoimmunity, impaired vaccine responses, and reduced immunosurveillance in cancer. Recent findings demonstrate that time-of-day-dependent administration of vaccines and immunotherapies, including checkpoint inhibitors, can significantly influence clinical efficacy and immune outcomes. Understanding the temporal orchestration of adaptive immunity thus holds translational potential for optimizing therapeutic strategies, including chronotherapy and vaccination scheduling.

## 1 Introduction to circadian rhythms

Circadian rhythms are mechanisms developed by organisms to adapt to the changing conditions of day and night caused by the Earth’s rotation (1). These intrinsic cycles, lasting 24 hours, regulate functions that vary throughout the day, such as sleep, temperature changes, metabolism, hormonal activity, regeneration and immune responses (2–4).

**Figure 1:**
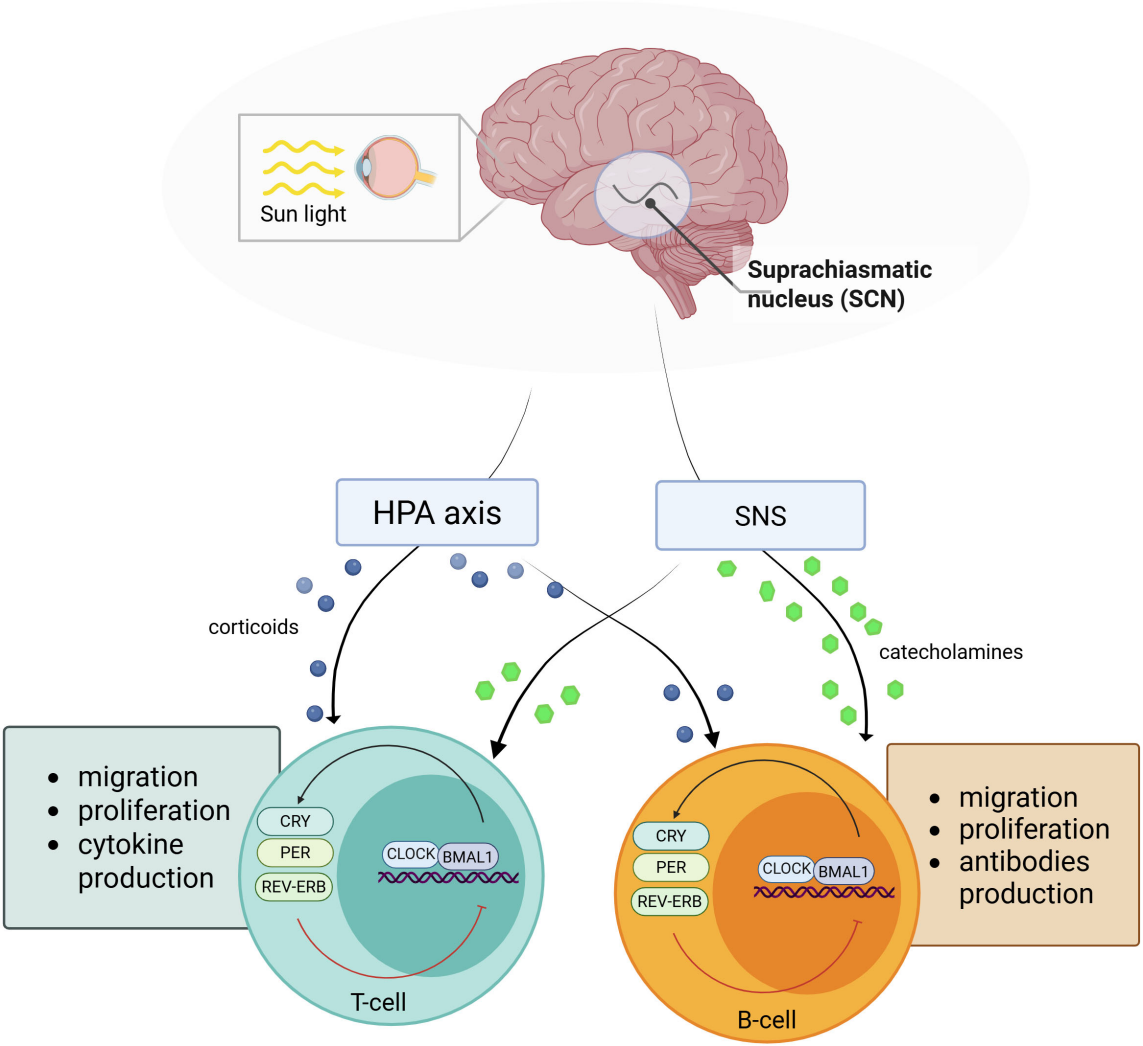
Interaction between the central circadian clock and peripheral clocks in T and B lymphocytes. Light signals perceived by the retina synchronize the central circadian clock located in the suprachiasmatic nucleus (SCN). The SCN coordinates rhythmic outputs via the hypothalamic–pituitary–adrenal (HPA) axis and the sympathetic nervous system (SNS). Neurohormonal signals, including glucocorticoids and catecholamines, entrain peripheral clocks in T and B lymphocytes, regulating the expression of core clock genes (CLOCK, BMAL1, PER, CRY, REV-ERB). This central–peripheral clock coupling controls key immune functions, including migration, proliferation, cytokine production (T cells), and antibody production (B cells). Created in BioRender. Sz, A. (2025) https://BioRender.com/wrun7z1.

Circadian rhythms are controlled by central and peripheral mechanisms. In mammals, the central clock is located in the suprachiasmatic nucleus (SCN) (5). This area integrates external stimuli, such as light, temperature, feeding times, physical activity, and social interactions, along with internal signals like hormones and metabolic cues with signals from the internal clock (6). In addition to the central mechanisms, there also exists a peripheral clock present in nearly all cells in the body. Although these peripheral clocks are dependent on the central clock, they have ability to independently regulate rhythmic gene expression (7, 8) (**Figure 1**).

**Figure 2:**
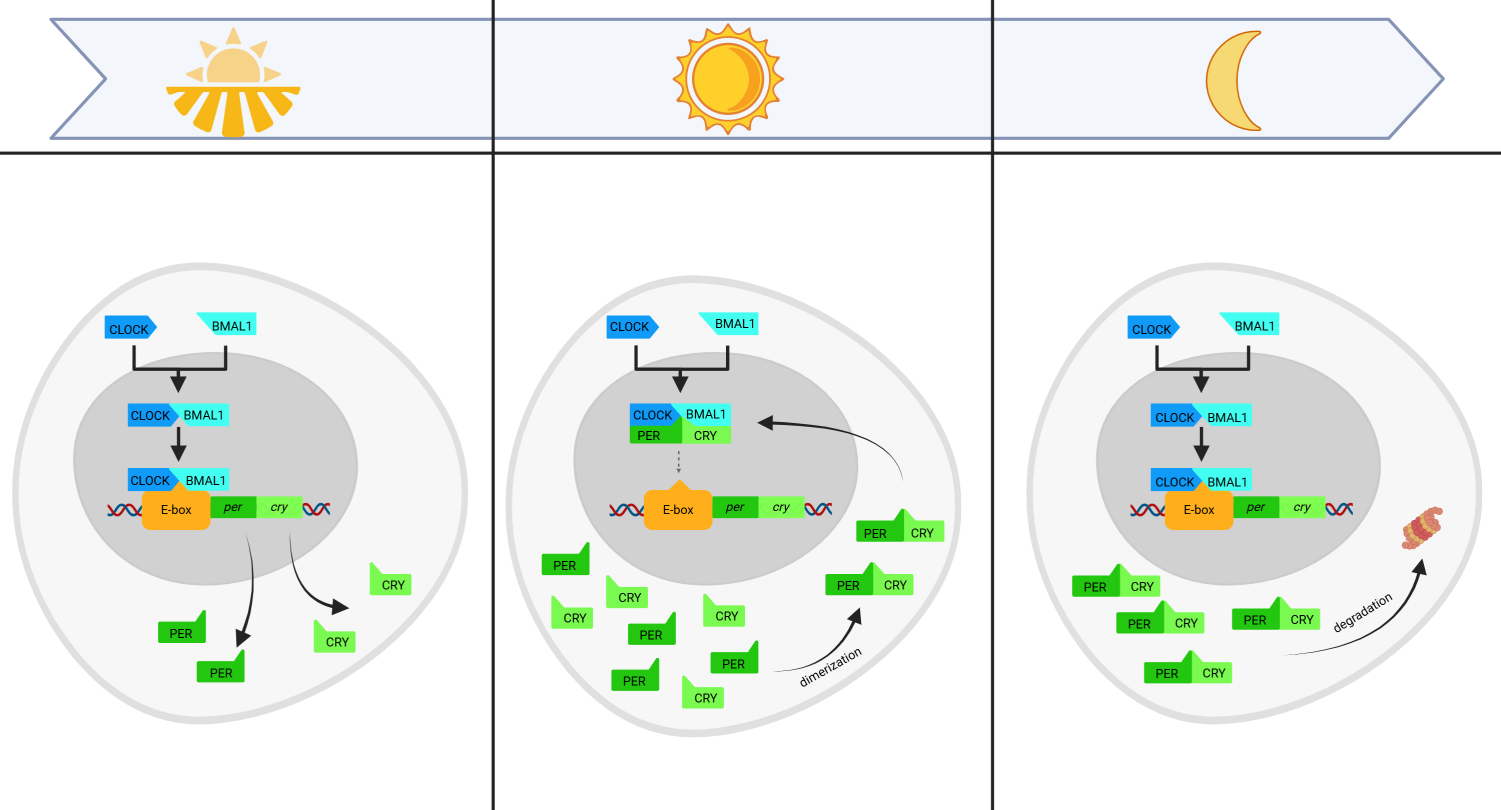
Feedback loop mechanism in circadian clock gene regulation. The diagram illustrates the oscillatory cycle of circadian clock gene regulation across a 24-hour period. Morning (left panel): the heterodimeric transcriptional activator CLOCK-BMAL1 binds to E-box promoter elements, inducing transcription of Period (Per) and Cryptochrome (Cry) genes. The translated PER and CRY proteins progressively accumulate in the cytoplasm. Daytime (middle panel): PER and CRY proteins dimerize and translocate into the nucleus, where they inhibit CLOCK-BMAL1-mediated transcription of their own genes, establishing a negative feedback loop. Nighttime (right panel): nuclear PER/CRY complexes undergo proteasomal degradation, relieving inhibition of CLOCK-BMAL1 and allowing a new cycle of Per and Cry transcription to begin. This self-sustained transcription–translation feedback loop underlies the ~24-hour circadian rhythm present in nearly all mammalian cells. Created in BioRender. Sz, A. (2025) https://BioRender.com/rox93ai.

The existence of circadian rhythms relies on specific “clock genes.” The first known clock gene was *period (per)*, discovered in Drosophila melanogaster (9), followed by the discovery of the *Clock* gene in mice (10). Since then, many other circadian-regulating genes, like *brain and muscle ARNT-like 1 (bmal1)* (11), and *cryptochrome (cry)* (12), have been identified. These genes exhibit oscillations that enable cyclic, 24-hour gene expression. The fundamental mechanism behind circadian rhythms is the feedback loop, which for the purposes of this review will be discussed on the example of the *clock* and *bmal1* genes, encoding the CLOCK and BMAL1 proteins, respectively. Together, these proteins form a complex that binds to a promoter region known as the E-box, thereby activating the transcription of the *per* and *cry*, which produce the PER and CRY. PER and CRY accumulate during the day and, upon reaching a threshold when they bind together forming a dimer complex, migrate to the nucleus. In the nucleus, this complex inhibits *per* and *cry* expression by interacting with CLOCK-BMAL1. The gradual degradation of PER and CRY - mainly through proteasome degradation and phosphorylation - leads to a decrease in their levels, allowing CLOCK-BMAL1 to bind with the E-box again, restarting the cycle (13–15) (**Figure 2**).

## 2 Circadian rhythms and the immune system

Research on circadian rhythms began in the 1950s and primarily focused on physiological processes such as sleep, body temperature, and metabolism (1). Over time, it became apparent that not only the nervous and hormonal systems, but also the immune system, operates in a rhythmic manner. A key turning point was the discovery in the 1990s of the central biological clock in the brain and the molecular mechanisms related to the expression of clock genes (16). This enabled the study of how biological rhythms regulate immune cell activity and function.

As molecular knowledge deepened in the early 21st century, it was observed that innate immune cells—such as macrophages, neutrophils, and dendritic cells-exhibit their own activity rhythms, independent of signals from the central clock. It became evident that the expression of many key genes involved in pathogen recognition, cytokine production, and leukocyte migration changes in a predictable manner over the course of a day (17, 18).

One of the breakthrough studies in this period, published by Silver et al. in 2012, demonstrated that macrophages, as the main cells responsible for pathogen recognition, phagocytosis, and elimination, exhibit increased phagocytic activity and heightened secretion of pro-inflammatory cytokines during the animal’s resting phase (i.e., the light phase in nocturnal mice). Notably, this phase was also associated with elevated expression of pattern recognition receptors, such as Toll-like receptor 9 (TLR9), which plays a key role in detecting unmethylated CpG motifs in microbial DNA. The upregulation of TLR9 during the resting phase likely enhances the sensitivity of macrophages to microbial components, facilitating more rapid and robust activation of downstream inflammatory signaling cascades (19). These findings suggest that the circadian regulation of macrophage activity during the resting phase enhances the initiation and amplification of immune responses.

The rhythmic expression of genes involved in neutrophil migration, along with the regulation of adhesion molecules and chemokines, was intensively studied between 2014 and 2018, primarily by research groups led by Matthias Nahrendorf and Filip Swirski. During the active phase, neutrophil blood count is higher, preparing the body for potential contact with pathogens during periods of increased mobility. In contrast, during the resting phase, their migration to tissues and lymphoid organs intensifies, where neutrophils perform protective and housekeeping functions. This migratory rhythm correlates with cyclical changes in the expression of adhesion molecules and chemokines that guide their movement, such as Intercellular Adhesion Molecule 1 (ICAM-1), Vascular Cell Adhesion Molecule 1 (VCAM-1), and selectins (E-selectin, P-selectin), which enable their adhesion to the endothelium, as well as chemokines, including C-X-C motif chemokine ligand 1 (CXCL1) and CXCL2, which recruit neutrophils via the C-X-C motif chemokine receptor 2 (CXCR2) and CXCL12, which acts through the CXCR4, directing their movement to tissues and lymphoid organs during the resting phase (20–23).

Experimental models using targeted modifications of core clock genes such as *bmal1* have provided direct evidence linking circadian gene expression to innate immune cell function. Disruption of this molecular clock leads to the loss of rhythmicity in cytokine production, dysregulation of the inflammatory response, and increased susceptibility to infections and the development of chronic inflammatory conditions. For example, mice with myeloid-specific *bmal1* deletion exhibit impaired control of bacterial infections and higher mortality in sepsis models, particularly when challenged during the resting phase. This confirmed that the circadian rhythm not only serves an organizational role in the immune system’s temporal activity but is also an active regulator of its efficiency and balance (24–26).

In recent years, there has been a growing interest in the impact of circadian rhythms on adaptive immunity, including T and B lymphocytes and the response to vaccinations. Studies have shown that the expression of surface molecules on lymphocytes, their proliferation, and cytokine production also undergo rhythmic regulation (27). Strikingly, although the development of a full adaptive immune response typically requires 1–2 weeks following antigen exposure, growing evidence suggests that a key factor determining the magnitude of the response is the time of day at which the exposure occurred. As these observations are emerging as clues potentially improving the effectiveness of vaccination and immunotherapy, a detailed understanding of the mechanisms by which circadian rhythms influence cells of the adaptive immune response is of great importance (28).

## 3 Circadian rhythms and trafficking

Circadian rhythms significantly regulate the migration of lymphocytes. In murine models, Cluster of Differentiation 4+ (CD4+), CD8+ T-cells and B lymphocytes exhibit rhythmic trafficking to lymph nodes, reaching peak numbers during the resting phase (29). Although direct evidence in humans is limited, peripheral blood studies suggest that lymphocyte counts fluctuate over the day, indicating possible circadian modulation of trafficking patterns, which may similarly involve lymphoid tissues (30). This process is primarily driven by the C-C Chemokine Receptor 7 (CCR7) present on lymphocytes, which mediates their entry into lymph nodes by binding to chemokines C-C Chemokine Ligand 19 (CCL19) and CCL21, secreted mainly by endothelial cells and dendritic cells. In contrast, lymphocyte egress is regulated by the Sphingosine-1-Phosphate Receptor 1 (S1PR1), which promotes the release of lymphocytes into efferent lymphatic vessels (31). Studies conducted on mice by Druzd et al. showed that the expression of CCR7 undergoes circadian oscillations, peaking at Zeitgeber Time 13 (ZT13) (early dark phase), coinciding with the maximal influx of lymphocytes. In CCR7-deficient mice, the rhythmicity of lymphocyte numbers is abolished (29). Additionally, studies using mice with targeted deletion of the *bmal1* gene in T and B lymphocytes demonstrated that clock genes, particularly *bmal1* and *clock*, are essential for the rhythmic expression of CCR7 and S1PR1, which control the timing of lymphocyte entry and egress from lymph nodes (22).

Other receptors involved in lymphocyte migration include CXCR4 and C-X3-C motif chemokine receptor 1 (CX3CR1). Naive and central memory T lymphocytes (both CD4^+^ and CD8^+^) exhibit circadian fluctuations in CXCR4 expression with their levels increasing during the active phase. This rise in CXCR4 expression enhances their homing to the bone marrow and lymph nodes specifically during periods of activity (32). Similarly, B lymphocytes (e.g., naive B subsets in the spleen) also exhibit circadian fluctuations of CXCR4 expression. Studies suggest that the morning rise in cortisol levels induces increased CXCR4 expression on B lymphocytes, resulting in a decrease in their numbers in the blood in favor of migration to the bone marrow and other lymphoid tissues (33).

**Figure 3:**
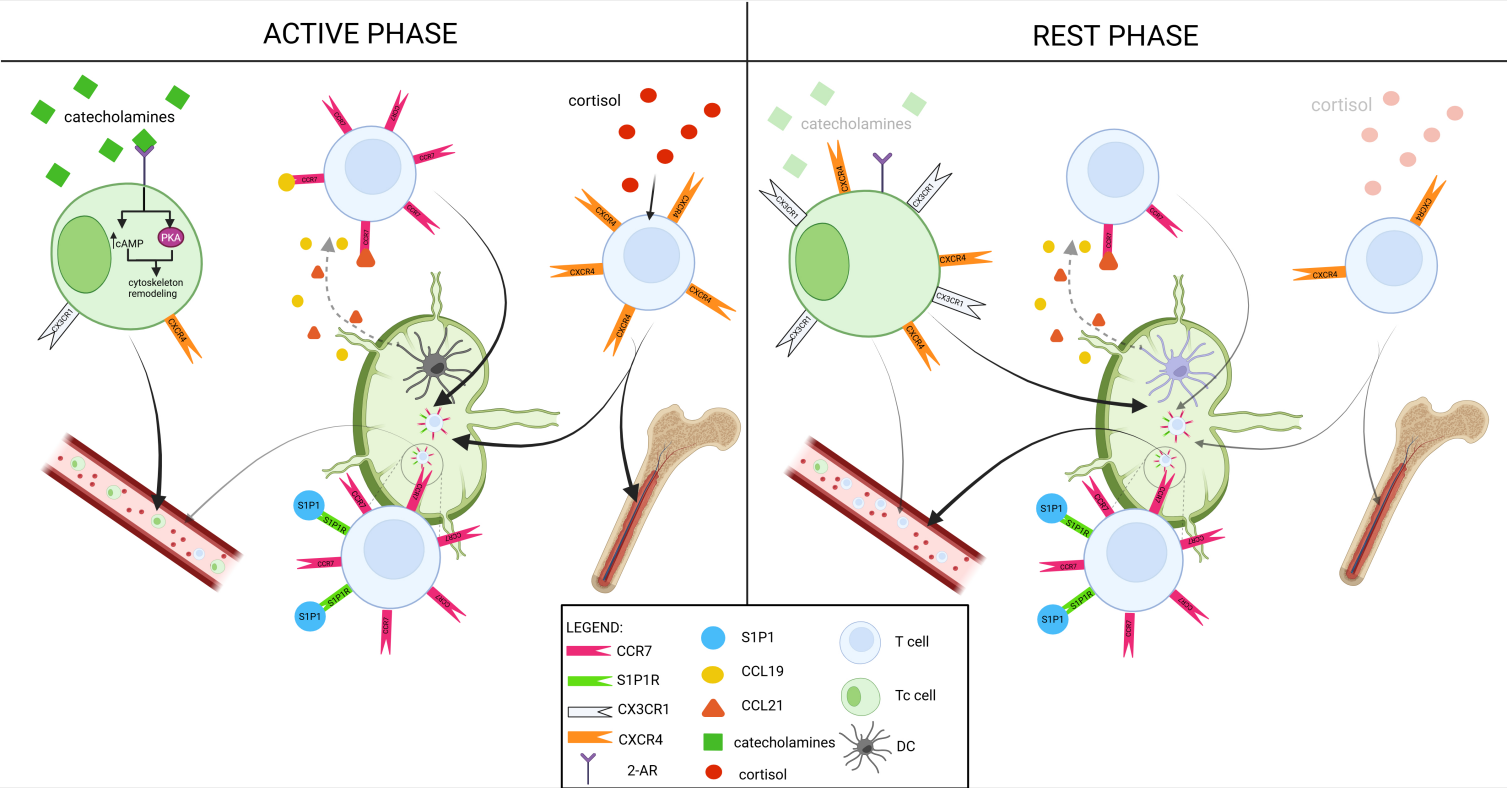
T-cells trafficking during active and rest phase. The schematic illustrates time-of-day–dependent regulation of lymphocyte migration controlled by chemokine receptors, adhesion molecules, and systemic signals. Active phase (left): elevated catecholamine release activates β2-adrenergic receptors (β2-AR) on lymphocytes, increasing intracellular cAMP and protein kinase A (PKA) signaling, which remodels the cytoskeleton and promotes lymphocyte egress into the bloodstream. CCR7 and S1P1 receptors coordinate T cell entry and exit from lymph nodes, while CXCR4 expression is upregulated, facilitating homing of naive and memory T cells to the bone marrow. CX3CR1-expressing effector T cells display enhanced endothelial adhesion. Cortisol further modulates receptor expression to optimize lymphocyte recirculation. Rest phase (right): reduced catecholamine signaling and altered glucocorticoid levels favor CCR7-mediated homing of lymphocytes to lymphoid tissues, increasing their abundance within lymph nodes. Dendritic cells (DCs) and chemokines (CCL19, CCL21) further orchestrate lymphocyte recruitment and retention during this period. This rhythmic redistribution ensures temporal optimization of immune surveillance, balancing peripheral circulation with tissue-specific immune readiness. Created in BioRender. Sz, A. (2025) https://BioRender.com/qzht58s.

In contrast, effector T lymphocytes (particularly the CD8+ subset) exhibit high and relatively stable expression of the CX3CR1 receptor, which facilitates their adhesion to the endothelium and binding to the vessel wall (34). During the active phase, the circadian peak in catecholamine release—mainly adrenaline and noradrenaline-plays a pivotal role in modulating lymphocyte trafficking. These catecholamines act on β2-adrenergic receptors (β2-AR) expressed on lymphocytes, initiating intracellular signaling cascades that elevate cAMP levels and activate protein kinase A (PKA). Activated PKA subsequently remodels the cytoskeleton, enhancing the cells’ migratory capacity. Importantly, catecholamine signaling also transiently downregulates the expression of chemokine receptors such as CXCR4 and CX3CR1 during the active phase, thereby reducing lymphocyte retention within lymphoid organs and promoting their release into the bloodstream (35) (**Figure 3**).

## 4 The role of circadian rhythms in T cell function

T cells, including CD4+ and CD8+ subsets, are essential components of the adaptive immune system. Their activation, proliferation, and effector functions are tightly regulated by numerous intrinsic and extrinsic factors (36). Recent evidence has established that circadian rhythms, orchestrated by the endogenous circadian clock, profoundly influence T cell development, migration, activation, and functional outcomes (37, 38).

### 4.1 Circadian clock machinery in T cells

T cells possess an intrinsic circadian clock governed by the core clock genes such as *bmal1, clock, per1, per2*, and *reverse erb alpha/beta (rev-erbα/β).* These genes modulate the expression of key components involved in T cell receptor (TCR) signaling, cellular metabolism (e.g., glycolysis, mammalian Target Of Rapamycin (mTOR) pathway), and cytokine and chemokine receptor expression (e.g., CD127, CXCR4), thereby influencing T cell processes in a time-of-day–dependent manner (39).

In CD8+ T cells, the clock proteins PER1 and PER2 play particularly important roles, with their rhythmic expression confirmed in both murine and human T cells (33, 40). These proteins synchronize T cell activity with physiological rhythms and modulate the timing of activation and effector differentiation. PER1 inhibits the Akt/mTORC1 pathway in naive CD4+ T cells, thereby promoting T helpers 2 (Th2) differentiation. PER2 defines a circadian phase of activation in CD8+ T cells-periods during the day when T cells are most immunoreactive-by regulating the rhythmic expression of surface receptors (e.g., TCR, Interleukin-2 Receptor (IL-2R), CD28, CXCR4), signaling kinases (Zeta-chain-associated protein kinase 70 (ZAP70), Akt, mTOR), and effector molecules (e.g., Interferon-γ (IFN-γ), perforin) (41). The critical role of PER1 and PER2 has been highlighted in studies by Yu et al. (2013) and Capelle et al. (2022), which showed that silencing *per1* and *per2* genes in mice leads to weakened T cell responses to antigens and an increased number of Th17 cells, potentially exacerbating autoimmune processes (42, 43).

**Figure 4:**
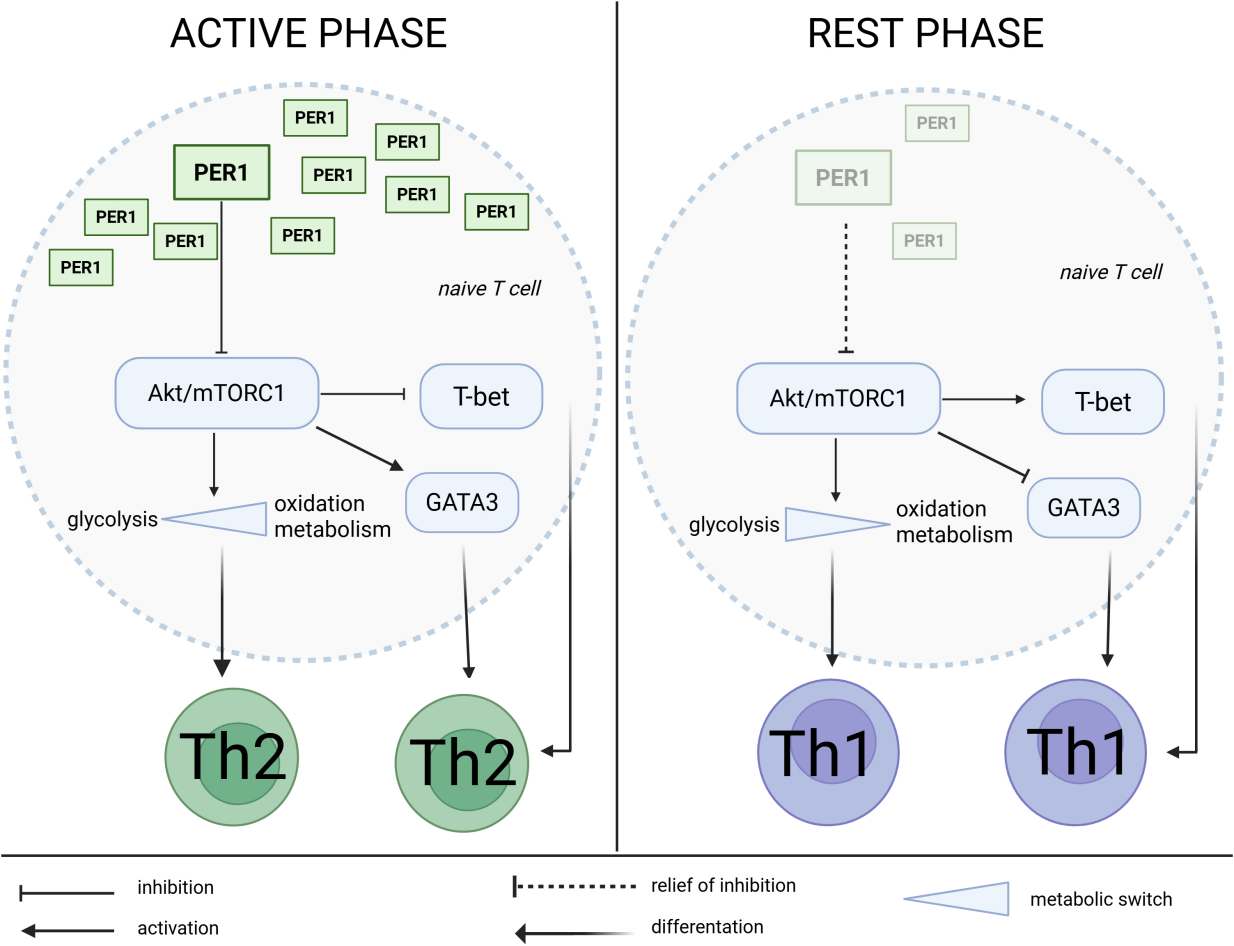
Circadian regulation of Th1/Th2 differentiation by PER1 and the Akt/mTORC1 pathway. During the active phase, elevated expression of the core clock component PER1 promotes activation of the Akt/mTORC1 pathway, enhancing glycolytic metabolism and the transcription factor GATA3, thereby favoring Th2 cell differentiation and type 2 immune responses. In contrast, during the rest phase, diminished PER1 expression reduces Akt/mTORC1 activity, leading to a metabolic shift toward oxidative phosphorylation and increased T-bet expression, which drives Th1 cell differentiation and type 1 immunity. This temporal regulation establishes a circadian gating mechanism that balances Th1 and Th2 polarization according to the time of day. Solid arrows represent activation; blunt-ended lines indicate inhibition; dashed lines denote relief of inhibition; and blue triangles depict metabolic switches between glycolysis and oxidative metabolism. Created in BioRender. Sz, A. (2025) https://BioRender.com/pdguh4k.

Beyond their temporal regulation of signaling components, PER proteins directly modulate the Akt/mTOR axis, which serves as a key metabolic checkpoint during T cell differentiation (43). PER1 suppresses Akt/mTORC1 activity in naïve CD4+ T cells, thereby shifting the balance away from glycolysis toward oxidative metabolism-a metabolic profile that favors Th2 differentiation (44). Reduced mTORC1 activity downstream of PER1 is associated with decreased expression of the transcription factor T-box expressed in T cell (T-bet), a driver of Th1 polarization, and enhanced GATA-binding protein 3 (GATA3) induction, which promotes Th2 lineage commitment (44, 45). Conversely, high mTORC1 activity, observed during PER1/2 suppression or circadian disruption, supports glycolytic metabolism, sustaining T-bet expression and effector Th1 cell generation (45). This, in turn, affects the balance between effector and regulatory T cell fates, including modulation of RAR-Related Orphan Receptor γt (RORγt), the master regulator of Th17 differentiation, which is particularly sensitive to mTOR-dependent signals (46). Collectively, these findings indicate that PER-mediated tuning of metabolic checkpoints links circadian timing to T cell fate decisions at the transcriptional level (**Figure 4**).

Although the molecular clock in T cells functions autonomously, it can be modulated by systemic signals to synchronize T cell function with the organism’s physiological rhythms. Glucocorticoids secreted in a circadian manner by the adrenal glands, under the control of the hypothalamic–pituitary–adrenal (HPA) axis, regulate the expression of *per1* and *rev-erbα* through binding of activated glucocorticoid receptors (GRs) to glucocorticoid response elements (GREs) in the promoters of clock genes (47–49). These glucocorticoids act to synchronize peripheral clocks, including those in T cells, with central circadian signals. Melatonin, secreted rhythmically by the pineal gland, influences the expression of clock genes such as *bmal1* and *per2* via Melatonin Receptor 1 (MT1) and MT2 receptors (50). Proinflammatory cytokines including IL-6, Tumor Necrosis Factor-α (TNF-α), and IL-1β also modulate clock gene expression, which may contribute to circadian disruption during chronic inflammation, for example by reducing *Bmal1* levels (51). Moreover, metabolic signals and body temperature can influence the circadian rhythm in T cells. Variations in energy substrate availability (e.g., glucose, fatty acids) affect the clock via Adenosine Monophosphate-Activated Protein Kinase (AMPK), mTOR, and Sirtuin 1 (SIRT1) pathways, which also regulate the molecular clock in T cells (52). Rhythmic changes in temperature regulate cold-inducible Ribonucleic Acid (RNA)-binding proteins (CIRP) modulates *Clock/Bmal1* expression (53).

### 4.2 Circadian influence on T cell development and differentiation

Circadian rhythms exert a significant impact on the differentiation of T cell subsets, particularly in the context of CD4+ T helper cells. The differentiation of Th1, Th2, Th17, and regulatory T cells (Tregs) is modulated by clock-controlled genes, which influence key signaling pathways such as Akt/mTOR and RORγt (54). As mentioned previously, the clock protein Per1 suppresses the Akt/mTORC1 pathway in naïve CD4+ T cells, thereby promoting Th2 differentiation and enhancing antibody-mediated responses to stress. Recent findings further demonstrate that Per1, induced by adrenergic or glucocorticoid signals, directly inhibits Th1 polarization via mTORC1 suppression-linking stress hormone signaling to Th1/Th2 balance through a CD4^+^ T cell-intrinsic mechanism (43).

Additionally, the transcription factor Nuclear Factor, Interleukin 3 Regulated (Nfil3) plays a pivotal role in the circadian regulation of Th17 cell differentiation. Its expression exhibits rhythmic diurnal oscillations that are in antiphase with the expression of the *rorc* gene, which encodes RORγt-the master regulator of Th17 differentiation. This antiphase relationship enables time-of-day-dependent modulation of Th17 responses, confining their activation to specific periods (42). Studies in *Nfil3*-deficient mice have shown that the absence of this factor leads to deregulated *rorc* expression, excessive Th17 cell differentiation, and heightened inflammatory responses patterns of Nfil3 and Rorc during the day modulating the magnitude of Th17 responses (55).

In Th17 cells, Nfil3 and RORγt exhibit robust antiphase oscillations that play a critical role in gating differentiation and effector function (56, 57). This regulatory axis is tightly linked to circadian modulation of chromatin accessibility at lineage-defining loci. During the rest phase, Nfil3 expression peaks, recruiting corepressors such as Histone Deacetylases (HDACs) and interacting with REV-ERBα to maintain a closed chromatin configuration at RORγt target enhancers (56). This represses transcription of Th17-associated genes, including *Il17a*, *Il23r*, and *Rorc* itself (42). In contrast, during the active phase, declining Nfil3 levels relieve this repression, coinciding with increased histone acetylation (e.g., Histone H3 lysine 27 acetylation (H3K27ac) and enhanced accessibility of RORγt-bound regulatory elements (58). Elevated RORγt is further activated by the acetyltransferases p300 (E1A-associated protein p300) and CBP (CREB-binding protein), which acetylate RORγt and promote recruitment of co-activating complexes (59). This temporal coordination ensures that Th17 responses are gated to specific circadian phases, likely optimizing host defense while limiting immunopathology (42) (**Table 1**).

**Table 1:** Temporal control of Th17 cell differentiation: antiphase oscillations of Nfil3 and RORγt across rest and active phases.

Feature	Rest phase	Active phase	Mechanism
Nfil3 expression	Peaks	Declines	High Nfil3 recruits corepressors (HDACs) and interacts with REV-ERBα
RORγt (encoded by Rorc) activity	Repressed	Activated	Low activity due to closed chromatin at target enhancers during rest; high activity during active phase
Chromatin accessibility	Closed at RORγt target enhancers	Open at RORγt target enhancers	Mediated by Nfil3 repression during rest and histone acetylation (H3K27ac) during active phase
Histone modifications	HDAC recruitment → deacetylation	Increased H3K27ac	Facilitates transcription of Th17 genes when Nfil3 declines
Th17-associated gene expression	Il17a, Il23r, Rorc → repressed	Il17a, Il23r, Rorc → expressed	Temporal gating restricts Th17 effector functions to active phase
Th17 differentiation/effector function	Suppressed	Enhanced	Ensures circadian control of immune response magnitude
Mechanistic outcome	Limits immunopathology	Optimizes host defense	Antiphase oscillation of Nfil3 and RORγt coordinates timing of Th17 responses

### 4.3 Circadian regulation of T cell activation and effector function

The timing of T cell activation via the T cell receptor (TCR) is influenced by circadian rhythms. Key signaling molecules involved in TCR activation, such as ZAP70, Akt, and mTOR, exhibit circadian oscillations in expression and activity. These rhythmic oscillations cause T cells to display peak responsiveness to antigenic stimulation during the organism’s active phase, thereby enabling a stronger immune response at times of greatest likelihood of pathogen exposure (60). For example, phosphorylation of ZAP70 peaks in mouse lymph nodes during the active phase, correlating with Akt/mTOR pathway activation and enhanced T cell proliferation (41). This regulation is supported by rhythmic glucocorticoid secretion, which peaks at dawn in humans and at dusk in nocturnal rodents (61). Glucocorticoids influence the expression of IL-7 receptor (CD127) and CXCR4, which are critical for T cell migration (49). The rhythmic expression of these receptors regulates the trafficking of T cells between the blood and secondary lymphoid organs (62), ensuring their presence in appropriate locations during the active phase and their “withdrawal” during sleep, when the risk of infection is lower.

### 4.4 Circadian disruption and immune dysregulation

CRD is defined as the perturbation of the intrinsic ~24-hour molecular clock machinery that governs the temporal regulation of gene expression and cellular physiology. This disruption arises from environmental challenges such as aberrant light-dark exposure, shift work, chronic jet lag, or from genetic alterations affecting core clock components, including c*lock, bmal1, per*, and *cry* genes. Such dysregulation leads to a loss of coherent oscillatory patterns in clock-controlled genes, thereby compromising downstream physiological pathways (63, 64). Experimental paradigms to investigate circadian disruption encompass rodent models exposed to shifted photoperiods, chronic phase advances or delays, and genetically engineered knockouts or mutants of clock genes. These models have elucidated mechanisms whereby circadian misalignment detrimentally affects cellular functions, including immune cell trafficking, metabolic homeostasis, and tissue repair processes (30).

CRD disturbs the balance between Th1/Th2 and Th17/Treg axes, favoring skewed differentiation toward Th2 and Th17 phenotypes (65). Chronic circadian dysregulation, as demonstrated in experimental models, may lead to hyperactivation of these subsets, thereby increasing the risk of allergic and autoimmune disorders (66). Yu et al. (2013) further reported that CRD promotes excessive generation of Th17 cells, which enhances inflammatory responses and susceptibility to autoimmunity (42). Moreover, it is hypothesized that CRD impairs the immunoregulatory function of Tregs, in part by reducing IL-10 production, which may disrupt immune homeostasis and negatively impact bone metabolism by promoting bone demineralization (67).

CRD have a significant impact on the functioning of the immune system, particularly affecting the activation, differentiation, and survival of T lymphocytes. Animal models have demonstrated that CRD leads to impaired T cell activation, reduced cytokine production (e.g., IFN-γ, IL-2), decreased proliferation, and diminished capacity of T cells to respond to antigens. For instance, deletion of the *bmal1* gene in T cells disrupts their rhythmic activity, resulting in reduced antigen responsiveness and diminished antiviral and antitumor immunity (41).

CRD negatively affects the differentiation and maintenance of memory T cells through several interconnected molecular and metabolic mechanisms. core clock genes, such as *bmal1* and *clock*, regulate the rhythmic expression of genes crucial for the survival and maturation of memory T cells, including B-cell lymphoma 2 (Bcl-2) and T-cell factor 7 (Tcf7). Deregulation of these genes leads to weakened immune responses and reduced memory T cell populations (68). Concurrently, disturbances in the rhythmic activity of the mTOR pathway hinder the metabolic shift from glycolysis (typical of effector T cells) to oxidative phosphorylation, which is essential for memory T cell survival (69). Additionally, CRD decreases the expression of cytokine receptors critical for memory T cell maintenance, such as IL-7Rα (CD127) and IL-15R, promoting premature apoptosis (68, 70).

Impaired T cell function due to disrupted gene rhythmicity caused by CRD is particularly evident in tumor models (71). In many cancers, the tumor microenvironment shows dysregulated expression of immune-checkpoint molecules, including Programmed cell death protein 1 (PD-1), Cytotoxic T-Lymphocyte Antigen 4 (CTLA-4), T-cell Immunoglobulin and Mucin-domain containing-3 (TIM-3), and Lymphocyte Activation Gene-3 (LAG-3) (72, 73). Overexpression of these inhibitory receptors may lead to T cell anergy, which in the context of cancer facilitates immune escape and weakens antitumor immune responses (54, 72, 73).

Furthermore, the circadian rhythm regulates the expression of chemokine receptors (e.g., CXCR3, CCR7) and adhesion molecules (e.g., ICAM-1, VCAM-1), which govern T cell migration to lymph nodes and inflamed tissues. CRD disrupts this process, resulting in asynchronous localization of effector T cells, potentially leading to their deficiency at sites of infection or tumor growth and consequently impairing local immune responses (29, 74).

## 5 The role of circadian rhythms in B cell function

The role of circadian rhythms in the development and function of B lymphocytes is increasingly recognized, with core clock genes such as *bmal1* playing a pivotal role in maintaining immune homeostasis. *bmal1* deficiency has been shown to impair B cell development, leading to reduced B cell numbers in the blood and spleen (68). However, adoptive transfer studies suggest that this effect is not mediated by the intrinsic circadian machinery of B cells, but rather by components of the bone marrow microenvironment, where *bmal1* regulates B cell differentiation (75).

Bone marrow stromal cells (BMSCs) create a hematopoietic niche that supports the survival and differentiation of B cell progenitors (76). In these cells, *bmal1* regulates the expression of essential growth factors, such as IL-7, which is critical for early B cell survival and maturation. Disruption of IL-7 expression rhythms due to *bmal1* loss negatively impacts lymphopoiesis (77). Osteoblasts, in addition to their role in bone formation, contribute to the hematopoietic stem cell (HSC) niche and the development of downstream lineages, including B cells (78). *Bmal1* activity in osteoblasts modulates the production of chemokines such as CXCL12, which direct the migration and positioning of B cell precursors within the marrow. *Bmal1* deficiency may impair this function, leading to niche disorganization and reduced interactions between B cells and supportive factors (79).

Endothelial cells of the bone marrow vasculature further regulate access to nutrients, cytokines, and control vascular permeability (80). The rhythmic activity of *bmal1* in these cells governs the expression of adhesion molecules and factors critical for B cell trafficking between marrow niches. Thus, circadian disruption may interfere with proper B cell localization and maturation (22). In contrast, B cell-specific deletion of *bmal1* does not significantly affect B cell differentiation or function, underscoring the central role of the microenvironment in circadian regulation of B lymphopoiesis (81, 82).

Circadian disturbances, such as those experienced during shift work, also critically impact the immunoregulatory function of B cells, particularly the B10 subset (83). B10 cells suppress CD4^+^ T cell proliferation and promote the differentiation of type 1 regulatory T cells (Tr1) through the secretion of anti-inflammatory IL-10 (33). Studies by Wang et al. demonstrated that shift workers exhibit significantly reduced IL-10 production despite unchanged B10 cell counts. This effect was linked to increased expression of the circadian regulator gene *clock* in B10 cells under conditions of circadian disruption. *Clock* expression was inversely correlated with IL-10 levels. Such dysregulation impairs the ability of B10 cells to suppress CD4^+^ T cell proliferation and Tr1 induction, suggesting that circadian misalignment contributes to immunological dysfunction (83).

Beyond regulatory functions, circadian rhythm disruption increases susceptibility to autoimmunity by affecting other B cell subsets. Mice deficient in *cry1* and *cry2* develop an autoimmune phenotype resembling features of human systemic lupus erythematosus (SLE) (84). This phenotype is marked by elevated serum IgG, presence of autoantibodies, and immune complex deposition in the kidneys and lungs. These mice show altered B cell populations, including reduced pre-B cells in the bone marrow and increased mature recirculating B cells and peritoneal B2 cells (84). Enhanced B cell receptor (BCR) signaling, demonstrated by increased tyrosine phosphorylation of downstream signaling proteins, suggests hyperactivation of the BCR pathway as a mechanism promoting autoimmunity. Furthermore, the absence of CRY proteins leads to downregulation of the Complement Component 1q (C1q), whose deficiency is strongly associated with the pathogenesis of SLE (84).

Although these findings were derived from murine models, they carry important clinical implications. Circadian rhythm disturbances in humans have been associated with an increased incidence of autoimmune diseases, including rheumatoid arthritis and SLE (85). Mechanisms identified in mice-such as BCR pathway overactivation, shifts in B cell subset distribution, and C1q deficiency-may similarly occur in humans, particularly those chronically exposed to circadian disruption.

In summary, the biological clock represents a critical regulator of B cell development, migration, and immune function. Circadian rhythm disturbances not only impair immune homeostasis but also increase vulnerability to autoimmune disorders, highlighting the potential of chronotherapy as a strategy to modulate B cell-mediated immunity.

## 6 The importance of circadian rhythms in the context of vaccination

Circadian rhythms regulate various physiological processes, including immune responses, and play a significant role in vaccination efficacy. Studies have shown that the timing of vaccination influences immune responses, particularly T cell activation and cytokine production. In a murine model, Nobis, Dubeau-Laramée et al. (2019) demonstrated that administering an experimental vaccine—comprising ovalbumin (OVA) as a model antigen and the adjuvant poly(I:C)—during the animals’ active phase elicited significantly greater T cell expansion and cytokine production compared to vaccination during the rest phase (e.g., midnight), underscoring the critical role of circadian timing in modulating adaptive immune responses (41). These findings emphasize the importance of aligning vaccination timing with the body’s circadian rhythm to enhance vaccine efficacy.

Several studies have explored how vaccination timing affects immune responses in different populations. For example, an analysis of influenza vaccination found that in older adults, vaccination in the morning or afternoon influenced antibody titers, while no such effect was observed in younger individuals (86). This highlights the need for further studies to consider vaccination timing when evaluating vaccine effectiveness (87, 88). In contrast, a study on young adults receiving the Pfizer Coronavirus Disease 2019 (COVID-19) vaccine found no significant differences in antibody responses or adverse reactions between morning and afternoon vaccination (89, 90).

Vaccination timing has shown to impact immune responses in specific subgroups, such as the elderly and immunocompromised populations. A study on hepatitis B vaccination in shift workers found that shift workers had a higher risk of vaccine response failure, suggesting that work schedules may interfere with immune function (91). Similarly, in kidney transplant recipients, morning vaccination was associated with better immune responses, as delays in the second dose decreased seroconversion odds (92).

In children, vaccination time also influences outcomes. A study on varicella vaccination in children under six years old found that vaccination in the late morning to afternoon was associated with the lowest infection rates, following a sinusoidal pattern (93). Moreover, a study on COVID-19 vaccination found that breakthrough infection rates varied with vaccination timing, with late morning to early afternoon vaccinations showing the lowest rates. This pattern was most pronounced in younger (<20 years) and older (>50 years) individuals, and morning vaccinations were linked to reduced COVID-19-related hospitalizations (94).

In conclusion, there is an increasing body of evidence that circadian rhythms modulate immune responses to vaccination, and adjusting vaccination timing to align with these rhythms could enhance vaccine effectiveness, especially in vulnerable populations such as the elderly, children, and immunocompromised individuals.

## 7 Circadian rhythms and autoimmune diseases

### 7.1 Role of circadian rhythms in autoimmune diseases

Circadian rhythms are integral in maintaining immune homeostasis, and their disruption is increasingly recognized as a contributing factor in the pathogenesis of autoimmune diseases (84). The interplay between circadian rhythms and immune responses has profound implications for the onset and progression of conditions like rheumatoid arthritis (RA), systemic lupus erythematosus (SLE), celiac disease (CeD), ulcerative colitis (UC), autoimmune thyroiditis (AIT), and multiple sclerosis (MS).

#### 7.1.1 Rheumatoid arthritis

Rheumatoid arthritis is a chronic autoimmune disorder marked by persistent synovial inflammation, causing progressive joint damage, pain, and disability. It affects about 1% of the global population, occurring two to three times more often in women, and imposes a major burden on quality of life and healthcare systems (95). In RA, CRD are linked to immune dysregulation, particularly involving key circadian rhythm genes (CRGs) like NFIL3, which are upregulated in RA and correlate with pro-inflammatory pathways such as TNF-α/NF-κB, IL-17, IL-6, IL-1β, and Matrix Metalloproteinase 3 (MMP3). These pathways promote inflammation and suggest that CRGs could be targeted therapeutically to restore immune homeostasis (96, 97). Additionally, melatonin, a critical circadian regulator, has shown potential in alleviating RA symptoms. It modulates circadian genes (PER2, CRY2), reduces inflammation, and inhibits pro-inflammatory cytokines (IL-6, TNF-α), highlighting its therapeutic potential in RA-ILD (98, 99). Furthermore, loss of *bmal1* in fibroblast-like synoviocytes (FLS) in RA reduces arthritis susceptibility, emphasizing the circadian clock’s role in modulating inflammation and joint degradation (100). Chronobiological therapies that target these disruptions could help improve immune regulation in RA (101).

#### 7.1.2 Systemic lupus erythematosus

SLE is a chronic, multisystem autoimmune disorder associated with significant morbidity and mortality, primarily due to involvement of the kidneys, skin, joints, and central nervous system (102). Circadian disruptions are central to the progression of SLE. The circadian gene *bmal1* is pivotal in neutrophil function, and its deficiency in lupus mouse models leads to increased autoantibody production and immune complex deposition, underscoring the contribution of circadian disruption to SLE pathogenesis (103). In human patients, reduced *bmal1* expression correlates with higher disease activity, emphasizing its role in SLE (103). Additionally, circadian rhythm disruption is closely linked to lupus flare-ups, with a flare risk score serving as an effective predictor of disease exacerbation (85). Mendelian randomization analysis has shown an inverse causal relationship between CRD and SLE, with a positive relationship to glomerular disorders, highlighting CRD’s influence on lupus nephritis (85). Gene expression profiling in lupus nephritis revealed a circadian signature that distinguishes disease subtypes, with B-cell- and T-cell-dominated patterns tied to disease severity (104).

#### 7.1.3 Celiac disease

Celiac disease is an autoimmune disorder induced by gluten, leading to small intestinal inflammation, malabsorption, and broad systemic effects that impair nutrition and quality of life (105). In CeD, circadian rhythm disruptions are associated with inflammation. A study examining the expression of circadian clock genes in leukocytes from newly diagnosed, untreated CeD patients found significant reductions in the expression of *clock, bmal1, cry2, per1*, and *per2*, while *cry1* levels remained unchanged (106). Chronic inflammation in celiac disease elevates pro-inflammatory cytokines (TNF-α, IL-6, IFN-γ), which directly modulate circadian clock gene expression by affecting transcription and protein stability. This leads to desynchronization of the biological clock, exacerbating intestinal inflammation and creating a feedback loop between inflammation and circadian disruption. Such desynchronization also impairs metabolic regulation, worsening symptoms like fatigue, malabsorption, and weight management (107). Additionally, circadian disruption may compromise immune function, increasing infection susceptibility and autoimmunity. Similar mechanisms are observed in other autoimmune diseases, including inflammatory bowel disease, where reduced circadian gene expression supports the role of circadian dysregulation as a pathogenic factor influencing disease progression and severity (106).

#### 7.1.4 Ulcerative colitis

UC is a chronic inflammatory disease of the colon with complex immune dysregulation and a risk of serious complications, including colorectal cancer, making it a critical focus of medical research (108). UC also exhibits circadian rhythm disturbances that influence disease pathogenesis. The circadian gene *per2* is significantly reduced in CD4+ T cells from inflamed mucosa and peripheral blood of UC patients. *Per2* suppresses Th1-driven inflammation by downregulating A Disintegrin And Metalloprotease12 (ADAM12), which promotes IFN-γ production. Restoration of *per2* expression during remission indicates its role in regulating immune balance, making it a promising therapeutic target for modulating CD4+ T cell differentiation and inflammation in UC (40).

#### 7.1.5 Autoimmune thyroiditis

AIT is a prevalent autoimmune disorder characterized by chronic inflammation of the thyroid gland, leading to thyroid dysfunction and significant metabolic disturbances (109). In the study by Fu, Fan et al. (2023), patients with AIT showed significantly reduced expression of the circadian clock genes *bmal1* and *per2* in thyroid tissue. This reduction was inversely correlated with levels of thyroid autoantibodies, suggesting a link between circadian disruption and enhanced autoimmunity (110). In a murine model of experimental autoimmune thyroiditis, diurnal oscillations of pro-inflammatory cytokines (TNF-α, IFN-γ) and anti-thyroglobulin antibodies were observed. Moreover, light-shift-induced circadian disruption intensified cytokine and autoantibody production, worsening disease severity. These findings indicate that chronic circadian misalignment may amplify inflammatory responses in AIT, forming a vicious cycle between circadian disruption and autoimmunity. Maintaining stable circadian rhythms may thus represent a potential therapeutic strategy for autoimmune thyroid disorders (110).

#### 7.1.6 Multiple sclerosis

Multiple sclerosis is a chronic autoimmune disease of the central nervous system characterized by neuroinflammation and demyelination, making it a leading cause of neurological disability in young adults (111). In MS, circadian rhythms regulate immune cell activation and migration (112). In the experimental autoimmune encephalomyelitis (EAE), a mouse model of MS, circadian disruptions in immune cells are observed, impacting behavior and immune responses. Loss of *bmal1* in myeloid cells creates an inflammatory environment in the central nervous system (CNS), increasing IL-1β-secreting monocytes and promoting the expansion of pathogenic IL-17+/IFN-γ+ T cells. This underscores the role of circadian rhythms in immune crosstalk during autoimmune diseases (112). Chronic inflammation in MS further disrupts circadian gene expression in immune cells, with T cells in EAE mice exhibiting altered circadian rhythms independent of central clock function, suggesting that peripheral immune cells are directly affected by inflammation (113).

## 8 Therapeutic importance of circadian rhythms in immunotherapy

Circadian rhythms are gaining increasing attention for their potential to enhance the effectiveness of immunotherapies. Immune checkpoint inhibitors (ICIs), which block regulatory proteins like PD-1, PD-L1, or CTLA-4 to enhance T-cell activation and enable immune responses against cancer, are significantly influenced by circadian rhythms (114). Studies indicate that the activity of cytotoxic CD8+ T cells, crucial for antitumor immunity, fluctuates throughout the day in alignment with circadian rhythms (115, 116). Administering ICIs during peak T-cell activity has been shown to enhance tumor reduction and improve clinical outcomes, highlighting the need to consider circadian timing in immunotherapy strategies (74, 117).

Recent research further underscores the importance of circadian rhythms in optimizing ICI administration. In a murine colorectal cancer model, variations in immune cell populations, particularly myeloid-derived suppressor cells (MDSCs) that express PD-L1, were found to follow circadian patterns (71). These immunosuppressive cells peak at specific times of the day, contributing to tumor immune evasion. Disruption of the epithelial cell clock altered cytokine and chemokine secretion, leading to increased inflammation and enhanced MDSC development. Administering anti-PD-L1 therapy during periods of peak MDSC abundance significantly improved treatment efficacy, resulting in 40–50% tumor growth inhibition and prolonged survival. These effects were accompanied by favorable changes in the tumor microenvironment, including reduced suppressive cell populations and increased activation of CD8+ T cells. These findings suggest that aligning immunotherapy with circadian rhythms can substantially enhance its therapeutic efficacy (116).

In addition to T cells and MDSCs, circadian rhythms also influence DCs, which are essential for initiating immune responses. DCs exhibit circadian trafficking to tumor-draining lymph nodes, with peak migration occurring during the active phase of the circadian cycle (115). In a study by Wang, Barnoud et al. (2023), it was demonstrated that administering cancer immunotherapy at times aligned with peak DCs activity significantly enhanced antitumor efficacy. Mice treated during periods of high DCs migration showed stronger CD8^+^ T cell activation, improved tumor control, and prolonged survival. These effects were abolished in mice lacking the core clock gene *bmal1* in DCs, confirming the role of circadian regulation. Moreover, human monocyte-derived DCs also showed time-dependent expression of clock and activation genes, suggesting translational relevance. This finding supports the notion that optimizing the timing of treatment could significantly improve the efficacy of cancer therapies (115).

The integration of circadian rhythms into immunotherapy has shown particular promise in brain cancer treatment. Circadian regulation of immune functions, including T-cell activity and tumor microenvironment dynamics, can be leveraged to maximize therapeutic efficacy. Aligning immunotherapy delivery with natural circadian peaks can optimize immune response while minimizing off-target toxicity, which may enhance both the effectiveness and safety of treatments (118, 119).

Clinical studies further support the potential benefits of synchronizing ICIs with circadian rhythms. A meta-analysis found that administering ICIs earlier in the day (defined variably across studies, with most using cut-off time around 4.00-4.30 PM) was associated with a 50% reduction in the risk of death and disease progression in patients with advanced cancers: non-small cell lung cancer (NSCLC), renal cell carcinoma (RCC), melanoma, urothelial carcinoma, esophageal squamous cell carcinoma (120). Similarly, studies in melanoma, metastatic squamous cell carcinoma and NSCLC patients suggest that early morning infusions of ICIs, such as nivolumab, are linked to improved overall survival and progression-free survival compared to afternoon doses (121–123). These findings suggest that chronomodulation of ICIs can significantly enhance patient outcomes, particularly in advanced stages of disease.

Further research is also exploring the potential of chronotherapy in optimizing ICI administration. For instance, a study in advanced melanoma patients revealed that scheduling the first few ICI infusions in the morning led to improved survival outcomes (124). Similarly, studies in metastatic RCC patients indicated that receiving ICIs earlier in the day improved progression-free survival, supporting the idea that ICI timing could impact clinical outcomes (125).

In addition to optimizing ICI administration timing, advances in chronogenetic therapies are showing promise. Researchers have engineered cell-based therapies, such as induced pluripotent stem cells, to release therapeutic levels of interleukin-1 receptor antagonist in sync with circadian rhythms, offering a potential strategy for inflammatory diseases like rheumatoid arthritis (126).

To date, only a limited number of clinical studies have systematically evaluated the influence of circadian timing on cancer therapy and immune modulation. These efforts reflect growing recognition of chrono-immunology’s translational relevance but also reveal significant methodological and conceptual inconsistencies across studies. **Table 2** summarizes representative ongoing and completed clinical trials exploring how treatment timing, light exposure, fasting, and circadian alignment affect immunotherapy efficacy, chemotherapy tolerance, and cancer-related symptoms. Together, these studies illustrate both the potential and the current limitations of clinical chrono-oncology.

**Table 2:** Current clinical trials investigating circadian rhythm modulation in immunotherapy.

Trial ID	Title	Phase	Cancer type	Intervention(s)	Start date	Completion date	Study type
NCT06418139	Association of Pembrolizumab Infusion Time and Efficacy in Patients With Non-metastatic Triple-negative Breast Cancer (TNBC) Treated With Neoadjuvant Chemotherapy and Immunotherapy (PEMCLOCK)	N.A.	Triple-negative Breast Cancer	Comparison of patient’s characteristics, toxicities, tumor response and EFS	2024-05	2026-09	Observational
NCT05988970	Impact of Circadian Rhythm on the Spread of Circulating Tumor Cells in Lung Cancer Patients (SLEEP_CTC)	N.A.	Lung cancer	CTC before and after treatment	2024-01-29	2025-09	Interventional
NCT04650490	SRS Timing With Immune Checkpoint Inhibition in Patients With Untreated Brain Metastases From Non-small Cell Lung Cancer (STICk-IM-NSCLC)	2	NSCLC with brain metastasis	Timing of stereotactic radiosurgery relative to immunotherapy	2023-03	2026-03	Interventional
NCT05737732	The Ambient Light Multiple Myeloma Study	N.A.	Multiple myeloma	Effect circadian effective vs ineffective lighting on patients receiving ASCT	2023-02-13	2027-06-30	Interventional
NCT05083416	Effect of Prolonged Nightly Fasting on Immunotherapy Outcomes in HNSCC - Role of Gut Microbiome	N.A.	Head and neck cancer	PNF vs no PNF effect on immunotherapy	2021-10-20	2024-02-08	Interventional
NCT04827446	Lighting Intervention for Cancer-related Fatigue	N.A.	Breast and prostate cancer, HSCT receivers	Effect of light levels on cancer related fatigue	2021-07-15	2023-03-09	Interventional
NCT04864405	Evaluating the Dose Timing (Morning vs Evening) of Endocrine Therapy and Its Effects on Tolerability and Compliance	4	Breast Cancer	Morning vs evening endocrine therapy	2021-06-30	2023-07-29	Interventional
NCT04669574	Assessing Sleep and Circadian Rhythms in Primary Brain Tumors Patients	N.A.	Primary brain tumor	Detect sleep disturbances in patients	2021-06-29	2024-12-09	Observational
NCT04401189	The Role of Circadian Rhythms in Cancer-Related Symptoms (CHRONO)	N.A.	Breast Cancer	Circadian rhythms and CRS before vs after treatment (surgery/chemotherapy)	2020-06-01	2023-06-30	Observational
NCT03217201	Systematic Light Exposure to Treat Cancer-Related Fatigue in Breast Cancer Patients	N.A.	Breast cancer	Effect of SLE on CRF	2018-01-25	2022-01-26	Interventional
NCT03196869	The Study of Effect of Chronomodulated Chemotherapy on the Dendritic Cells Subsets in the Treatment of Advanced Nasopharyngeal Cancer	2	Locally advanced HNSCC	Chrono-chemotherapy vs routine chemotherapy	2017-04-07	2022-08-12	Interventional
NCT05637580	Pathological Tumor and Lymph Node Responses After Neoadjuvant Immunochemotherapy in Initially-unresectable NSCLC	N.A.	NSCLC	Timing of drug administration	2019-05-20	2022-07-01	Observational
NCT02954809	Feasibility of Bright Light Therapy on Fatigue, Sleep and Circadian Activity Rhythms in Lung Cancer Survivors	N.A.	Lung cancer survivors	Morning bright light therapy vs dim light	2016-10	2018-01	Interventiomal
NCT02781792	Temozolomide Chronotherapy for High Grade Glioma	2	High grade glioma (II-IV)	TMZ morning vs evening administration	2016-08-11	2024-07-18	Interventional
NCT03955510	Abnormal Food Timing and Circadian Dyssynchrony in Alcohol Induced Colon Carcinogenesis (AFT)	N.A.	Colorectal Cancer	Effect of timing of food intake and alcohol use on susceptibility to colorectal cancer.	2016-07-31	2025-11-30	Interventional
NCT03205033	Melatonin as a Circadian Clock Regulator, Neuromodulator and Myelo-protector in Adjuvant Breast Cancer Chemotherapy	2	Breast Cancer	Melatonin 7 days before until 21 days after chemotherapy	2016-01	2017-01	Interventional
NCT02937519	Chronomodulated Chemotherapy Followed by Concurrent Chemo-radiotherapy With IMRT in the Treatment of Advanced Nasopharyngeal Cancer	2	Locally advanced nasopharyngeal carcinoma	Chrono-chemotherapy vs routine chemotherapy	2015-06	2018-06	Interventional
NCT02187315	Induction Chemotherapy Followed By Chrono-chemotherapy Concurrent With IMRT Of Locally Advanced NPC Clinical Study	2	Locally advanced nasopharyngeal carcinoma	Chrono-chemotherapy ves routine chemotherapy	2014-05	2019-12	Interventional
NCT00852228	Optimal Control of Liver Metastases From Colorectal Cancer With Cetuximab and Hepatic Artery Infusion of Chemotherapy (OPTILIV)	2	Liver metastases from colorectal cancer	Chronomodulated vs conventional chemotherapy	2008-07	2015-12	Interventional

## 9 Current gaps and controversies in chrono-immunology

Despite rapid progress in understanding circadian regulation of adaptive immunity, several important gaps and debates remain (127). The molecular links between core clock components (*Bmal1, Per, Cry*) and immune signaling pathways such as Akt/mTOR, NF-κB, and JAK–STAT are still incompletely understood, particularly in the context of different T and B cell subsets, tissue microenvironments, and disease states (128). It is also unclear how intrinsic lymphocyte clocks interact with systemic entrainment cues, including hormonal, metabolic, and neural signals (33). The balance between cell-autonomous rhythmicity and external control remains one of the central unresolved questions in the field (128).

Another area of uncertainty concerns the temporal regulation of immune memory and vaccine responses. While multiple studies show time-of-day–dependent effects on vaccination efficacy and immunotherapy outcomes, the underlying mechanisms, such as antigen presentation kinetics, T and B cell activation thresholds, and germinal center dynamics, remain incompletely characterized (33, 129). Moreover, results across human studies are heterogeneous, reflecting differences in experimental design, population chronotypes, and disease contexts (129). Finally, there is limited understanding of how chronic circadian disruption affects long-term immune adaptation, autoimmunity, and therapeutic responses (130).

Addressing these unresolved challenges will be crucial for translating chrono-immunological insights into clinically actionable strategies. Integrating mechanistic understanding with emerging chrono-omics technologies and individualized circadian profiling may bridge the gap between experimental discoveries and therapeutic application, paving the way for precision, time-tailored immunotherapies and vaccination strategies.

## 10 Conclusions and future research directions

Circadian rhythms play a critical role in regulating adaptive immunity by coordinating immune cell activity, gene expression, and the timing of immune responses to optimize host defense. Core clock genes such as *bmal1*, *per*, *cry*, and *rev-erb* govern rhythmic oscillations in T and B lymphocytes, regulating their differentiation, migration, activation, and survival. Disruption of these rhythms-whether intrinsic, such as genetic mutations, or extrinsic, such as altered light exposure or sleep disturbances-can lead to cytokine dysregulation, altered metabolic programming, and impaired immune cell trafficking (112, 131). These alterations compromise immune efficacy and tolerance, contributing to the progression of autoimmune and infectious diseases and enabling tumor immune escape (33).

Despite significant progress in the field of circadian biology and increasing interest in its relevance to adaptive immunity, many underlying mechanisms remain poorly understood and require further in-depth investigation. At the molecular level, it is crucial to determine the interactions between circadian regulators and epigenetic and metabolic pathways, as well as their impact on transcriptional profiles across specific immune cell subsets under both physiological and pathological conditions (132). Future studies should prioritize mapping these interactions using advanced technologies such as single-cell RNA sequencing, chronotranscriptomics, and temporal proteomics. The tissue microenvironment plays a pivotal role in shaping local immune responses and is itself subject to circadian regulation (133). Daily oscillations in oxygen availability, nutrient gradients, and cytokine expression may critically shape local immune dynamics and inflammatory states (37).

An additional priority is the development of circadian biomarkers to guide personalized chronotherapy. Rhythmic transcriptomic or proteomic signatures, hormonal oscillations, and immune cell trafficking patterns can provide objective measures of individual circadian phase. Integrating such biomarkers with clinical decision-making could enable optimized timing of vaccination, immune checkpoint blockade, or adoptive cell therapy, enhancing efficacy while minimizing toxicity (39, 41, 129). Incorporating patient chronotype assessment and wearable circadian monitoring into clinical trials represents an important next step toward precision immunotherapy.

In parallel the development of pharmacological agents targeting circadian regulators represents a novel and rapidly evolving therapeutic domain. Compounds such as REV-ERB agonists, ROR inhibitors, and melatonin analogs offer the ability to modulate core clock components, thereby influencing immune cell behavior in a temporally defined manner. Preclinical studies have demonstrated their potential in modulating inflammation and autoimmunity, and early-phase clinical trials are underway to evaluate their safety and efficacy in human populations (134, 135). However, their clinical translation will require careful evaluation of timing strategies, efficacy, and long-term safety.

Nevertheless, despite the promise of circadian-based interventions, critical gaps remain in our understanding of their long-term safety profiles. The novelty of these therapeutic modalities necessitates rigorous investigation into potential adverse effects, particularly when used in combination or over extended durations (136). Comprehensive safety assessments should therefore be integral to future clinical development. Moreover, environmental and behavioral disruptions-including shift work, exposure to artificial light at night, and irregular sleep patterns-pose substantial challenges to circadian alignment (137). These factors are increasingly recognized as contributors to immune dysregulation and heightened disease susceptibility (138). Interindividual variability in circadian phase and real-world behavioral influences must be considered when designing chronotherapy protocols and clinical trials.

In summary, integrating circadian biology into immunology not only enriches our mechanistic understanding but also opens new therapeutic avenues that harness the power of time to improve immune health. Future research should focus on a deeper mechanistic dissection of circadian–immune interactions, systematic biomarker discovery, and careful evaluation of therapeutic timing, efficacy, and safety.
